# Induction of sirtuin-1 signaling by resveratrol induces human chondrosarcoma cell apoptosis and exhibits antitumor activity

**DOI:** 10.1038/s41598-017-03635-7

**Published:** 2017-06-09

**Authors:** Sung-Chuan Chao, Ying-Ju Chen, Kuo-How Huang, Kuan-Lin Kuo, Ting-Hua Yang, Kuo-Yuan Huang, Ching-Chia Wang, Chih-Hsin Tang, Rong-Sen Yang, Shing-Hwa Liu

**Affiliations:** 1 Institute of Toxicology, College of Medicine, National Taiwan University, Taipei, Taiwan; 2 Department of Surgery, National Taiwan University Hospital Hsin-Chu Branch, Hsin-Chu, Taiwan; 3 Urology, National Taiwan University College of Medicine and Hospital, Taipei, Taiwan; 4 Department of Otolaryngology, National Taiwan University Hospital, Taipei, Taiwan; 5 Department of Orthopaedics, National Cheng Kung University Hospital, Tainan, Taiwan; 6 Department of Pediatrics, College of Medicine, National Taiwan University, Taipei, Taiwan; 7 Graduate Institute of Basic Medical Science, China Medical University, Taichung, Taiwan; 8 Department of Orthopaedics, College of Medicine, National Taiwan University, Taipei, Taiwan; 9 Department of Medical Research, China Medical University Hospital, China Medical University, Taichung, Taiwan

## Abstract

Chondrosarcoma is a malignant primary bone tumor. Sirtuin-1 (SIRT1), which is a member of sirtuin family, plays a dual role either in cancer promotion or suppression. There is no report about the role of SIRT1 in the human chondrosarcoma cells. Resveratrol is a potent activator of SIRT1. However, its effects on chondrosarcoma have not been extensively studied. Here, we investigated the role of SIRT1 induction by resveratrol in human chondrosarcoma cell growth and tumor progression. Resveratrol significantly decreased cell viability and induced cell apoptosis in human chondrosarcoma cells in a dose-dependent manner. The protein expression and activity of SIRT1 were activated after treatment with resveratrol. Resveratrol significantly inhibited NF-κB signaling by deacetylating the p65 subunit of NF-κB complex, which could be reversed by siRNA-SIRT1 transfection or deacetylation inhibitor MS-275. Resveratrol induced-apoptosis involved a caspase-3-mediated mechanism. Both siRNA-SIRT1 transfection and MS-275 significantly inhibited the resveratrol-induced caspase-3 cleavage and activity in human chondrosarcoma cells. Moreover, *in vivo* chondrosarcoma xenograft study revealed a dramatic reduction in tumor volume and the increased SIRT1 and cleaved caspase-3 expressions in tumors by resveratrol treatment. These results suggest that resveratrol induces chondrosarcoma cell apoptosis via a SIRT1-activated NF-κB deacetylation and exhibits anti-chondrosarcoma activity *in vivo*.

## Introduction

Chondrosarcoma is one of the most common primary bone tumors ranking after myeloma and osteosarcoma, accounting for approximately 20% of bone sarcomas and may occur at any age between 10 and 80 years^1^. Due to its poor response to both radiotherapy and chemotherapy, the management of chondrosarcoma faces a complicated challenge^2^. Clinically, surgical excision remains the choice of therapy for the majority of chondrosarcomas; however, the survival of high-grade chondrosarcoma patients is still poor after surgical therapy. Because of the absence of an effective adjuvant therapy, the prognosis of this mesenchymal malignancy is poor^3^, and the development of a novel and adequate remedy, therefore, is urgent.

Numerous naturally occurring substances are recognized as anti-oxidants, cancer preventive agents, or even as a cancer therapy drug. Resveratrol, first isolated from roots of white hellebore (*Veratrum grandiflorum O.Loes*.) in 1940, is a natural phenolic compound found in several plants such as peanuts, grapes, raspberries, and vegetables^4^. In 1997, resveratrol was first found to exert its anti-tumor effects^5^. Several studies have indicated that resveratrol inhibits several types of cancers, including colon cancer, breast cancer and lymphoma, through the regulation of various molecular targets^6–8^. Resveratrol has been suggested to possess heath functions, such as cardioprotection, neuroprotection, anti-oxidation, anti-inflammation, anti-infection, and anti-cancer^9–14^. It has been shown that the effects of resveratrol are associated with several cellular proteins, such as DNA polymerase, ribonucleotide reductase (RNR), and phosphatidylinositol 4,5-bisphosphate 3-kinase (PI3K)^15–17^. Moreover, resveratrol can activate the activities of sirtuins including surtuin-1 (SIRT1)^18, 19^. Resveratrol activates SIRT1 activation in a substrate sequence-dependent manner via deacetylation of a SF38-K23 peptide^20^. Resveratrol has also been shown to inhibit chondrosarcoma cell growth and induce apoptosis^21, 22^. However, the role of SIRT1 and the detailed effects and mechanisms of resveratrol on chondrosarcoma are still needed to be clarified.

Sirtuins belong to the class III histone deacetylases (HDACs) and are NAD^+^-dependent enzymes. There are seven isoforms of sirtuins in mammalian named SIRT1-7, which possess either histone deacetylase (SIRT1-3, SIRT5 and SIRT7) or monoribosyltransferase activity (SIRT4 and SIRT6)^23^. They are participated in a wide range of cellular processes and pathways with different cellular localization and molecular targets. SIRT1 is primarily a nuclear deacetylase and can communicate between the nucleus and cytoplasm under certain cases^24^. SIRT1 normally protects cells from oncogenic transformation; however, its enzymatic activity can also promote cancer growth by inactivation of proapoptotic factors^25, 26^. Among the sirtuins family, the involvement of SIRT1 in cancer has been most extensively studied, which results are decisively controversial and contradictory. SIRT1 plays a dual role either in cancer promotion or suppression, depending on different cellular contexts or its targets in specific signaling pathways or specific cancers^27^. It has been reported that SIRT1 deacetylates and inactivates hypoxia-inducible factor (HIF)-1α, thus suppresses the genes expression targeted by HIF-1α in certain tumors^28^. SIRT1 has been shown to inhibit pancreatic cancer cell proliferation and express oncogenic pancreatic adenocarcinoma-upregulated factors by inhibition of β-catenin and cyclin-D1^29^. SIRT1 has been found to suppress epithelial-mesenchymal transition (EMT) in HMLER breast cancer cells; reduced SIRT1 expression increases metastasis in nude mice^30^. Moreover, activation or overexpression of SIRT1 has been suggested to be involved in the regulation of apoptosis through deacetylation of p53 or other tumor suppressor proteins or FOXO transcriptions factors^31, 32^. Resveratrol has been reported to possess neuroprotective effect via SIRT1 activation in neurodegenerative disorders^33^. Li *et al*. also provided important information regarding the effect of resveratrol on osteosarcoma cells via SIRT1 activation^34^. Although the role of SIRT1 in cancers is still controversial, SIRT1 has been indicated to be a key target for resveratrol in several human tumor models^6^. To our knowledge, there is no report about the role of SIRT1 in the human chondrosarcoma cells. Therefore, we tried to evaluate the therapeutic effects of resveratrol via SIRT1 activation on chondrosarcoma cell growth and tumor progression using an *in vitro* cell culture model and an *in vivo* xenograft mouse model.

## Results

### Resveratrol induces cell apoptosis in human chondrosarcoma cells

**Figure 1:**
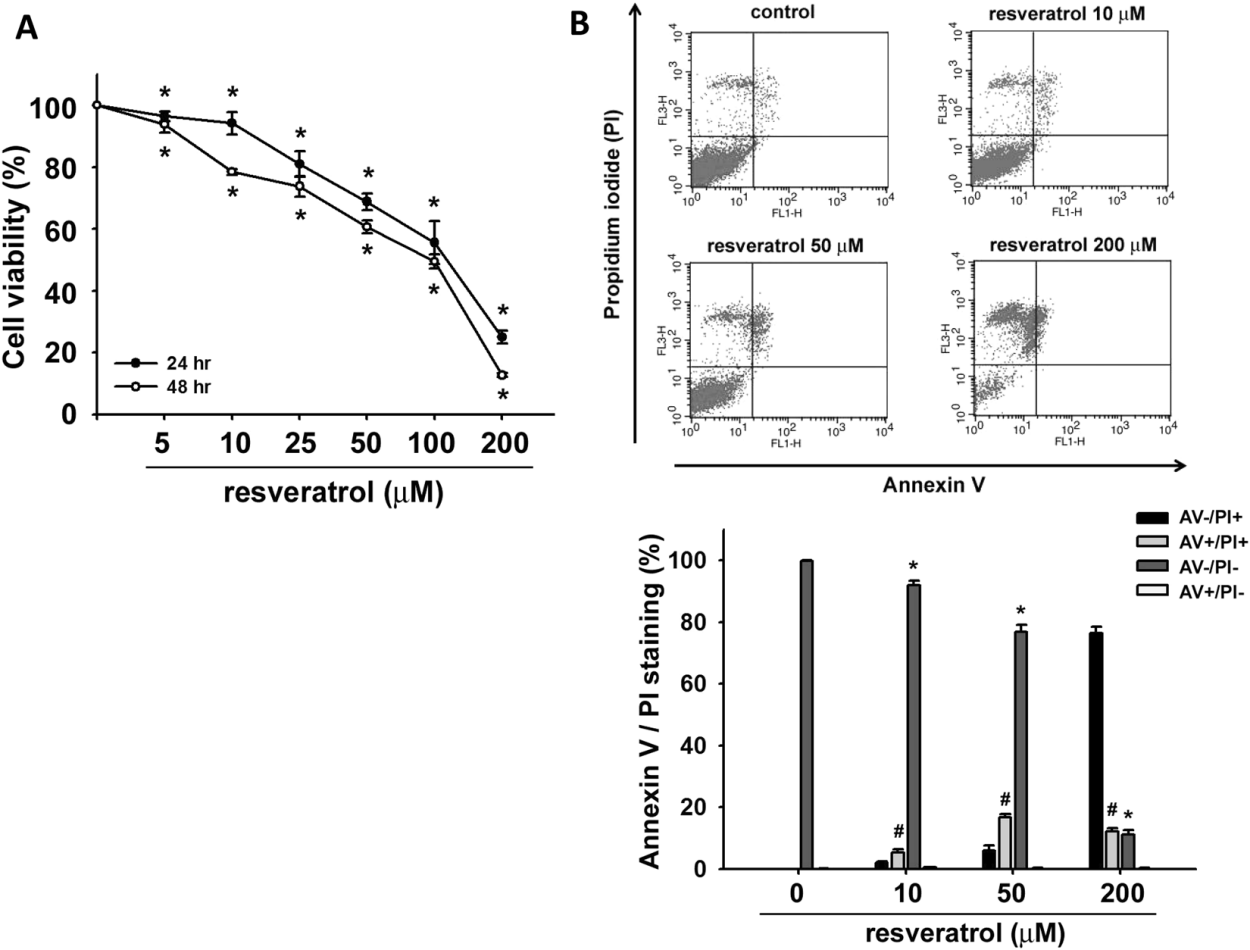
Effects of resveratrol on cell viability and apoptosis in human chondrosarcoma cells. (**A**) JJ012 cells were incubated with various concentrations of resveratrol (5–100 μM) for 24 and 48 h. The cell viability was examined by MTT assay. (**B**) JJ012 cells were incubated with various concentrations of resveratrol (10–200 μM) for 48 h and the percentage of apoptotic cells was analyzed by flow cytometry of annexin V/PI double staining. Results are presented as the mean ± SEM (n ≥ 4). *or ^#^ *P* < 0.05 as compared with control (*annexin^−^/PI^−^; ^#^annexin^+^/PI^+^).

To investigate the potential cell death of resveratrol in human chondrosarcoma cells, we first examined the effect of resveratrol on chondrosarcoma cell survival. As shown in Fig. 1A, treatment of chondrosarcoma cells with resveratrol significantly decreased cell viability in a dose-dependent manner. We next investigated whether resveratrol induced cell death through an apoptotic mechanism. Annexin V-PI double-labeling was used for the detection of PS externalization, a hallmark of early phase of apoptosis. As compared to vehicle-treated cells shown in Fig. 1B, a high proportion of annexin V+ labeling was observed in cells treated with resveratrol.

### Resveratrol induces the expression and activity of SIRT1

**Figure 2:**
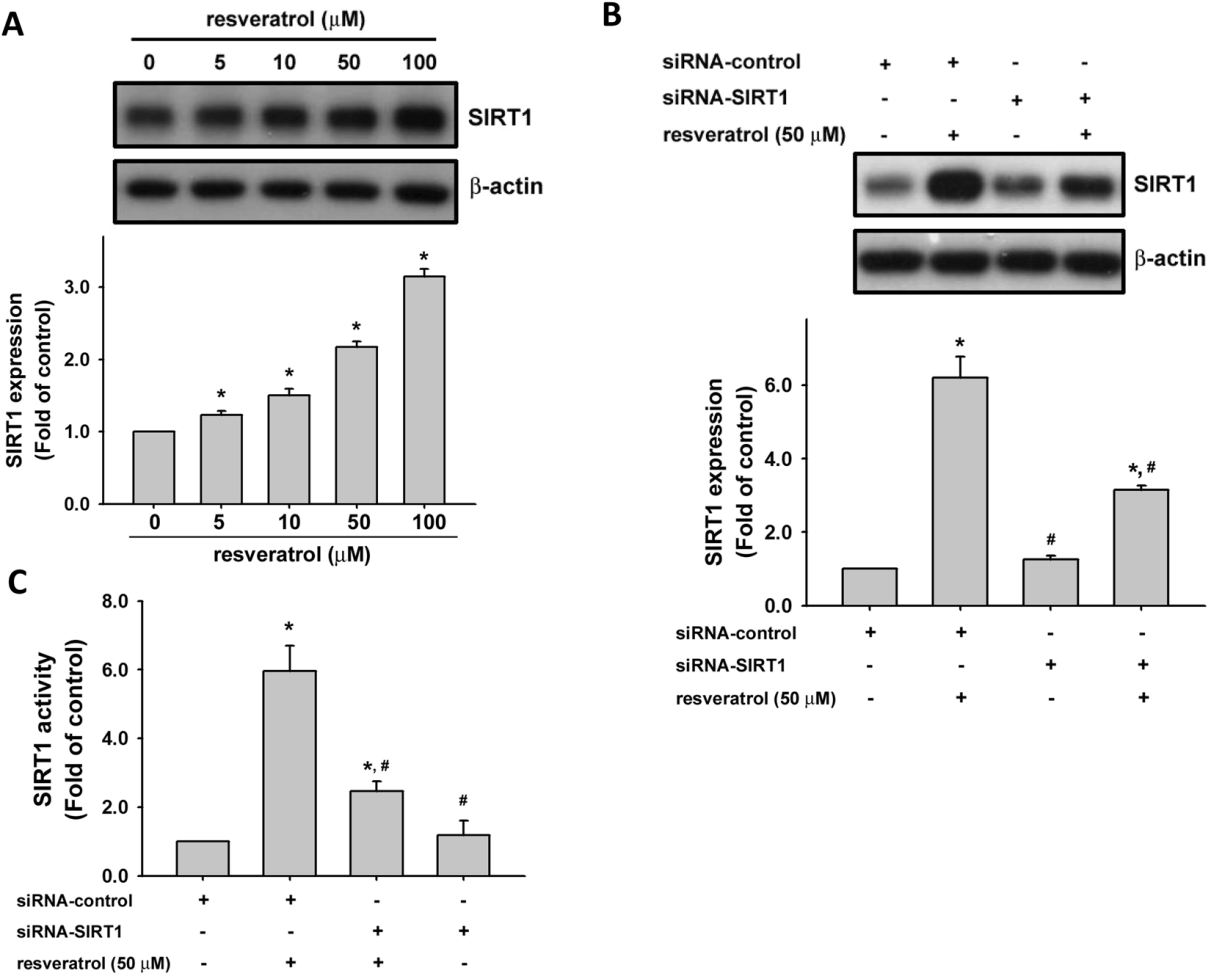
Involvement of SIRT1 activation in resveratrol-mediated chondrosarcoma cell apoptosis. (**A**) JJ012 cells were incubated with various doses of resveratrol (5–100 μM) for 24 h. (**B**) and (**C**) Cells were transfected with SIRT1 siRNA or control siRNA for 24 h with or without resveratrol treatment. The SIRT1 expression was examined by Western blot analysis (**B**). SIRT 1 activity was determined with SIRT1 Deacetylase Fluorometric Assay kit (**C**). Results are presented as the mean ± SEM (n ≥ 4). ^*^ *P* < 0.05 as compared with control; ^#^ *P* < 0.05 as compared with resveratrol-treated group.

SIRT1 possesses NAD^+^-dependent class III histone deacetylase activity. It has been suggested to be a key linking of gene between the modulation of cancer and aging^35^. We examined the effect of resveratrol on the expression and activity of SIRT1 in human chondrosarcoma cells. As shown in Fig. 2A, resveratrol markedly increased the protein expression of SIRT1 in a dose-dependent manner. To further confirm the effect of resveratrol on SIRT1, the SIRT1 knockdown by siRNA was used. Transfection of cells with SIRT1 siRNA specifically inhibited SIRT1 expression and SIRT1 activity in resveratrol-treated chondrosarcoma cells (Fig. 2B,C).

### Resveratrol attenuates NF-κB activation through the reduction of p65 acetylation

**Figure 3:**
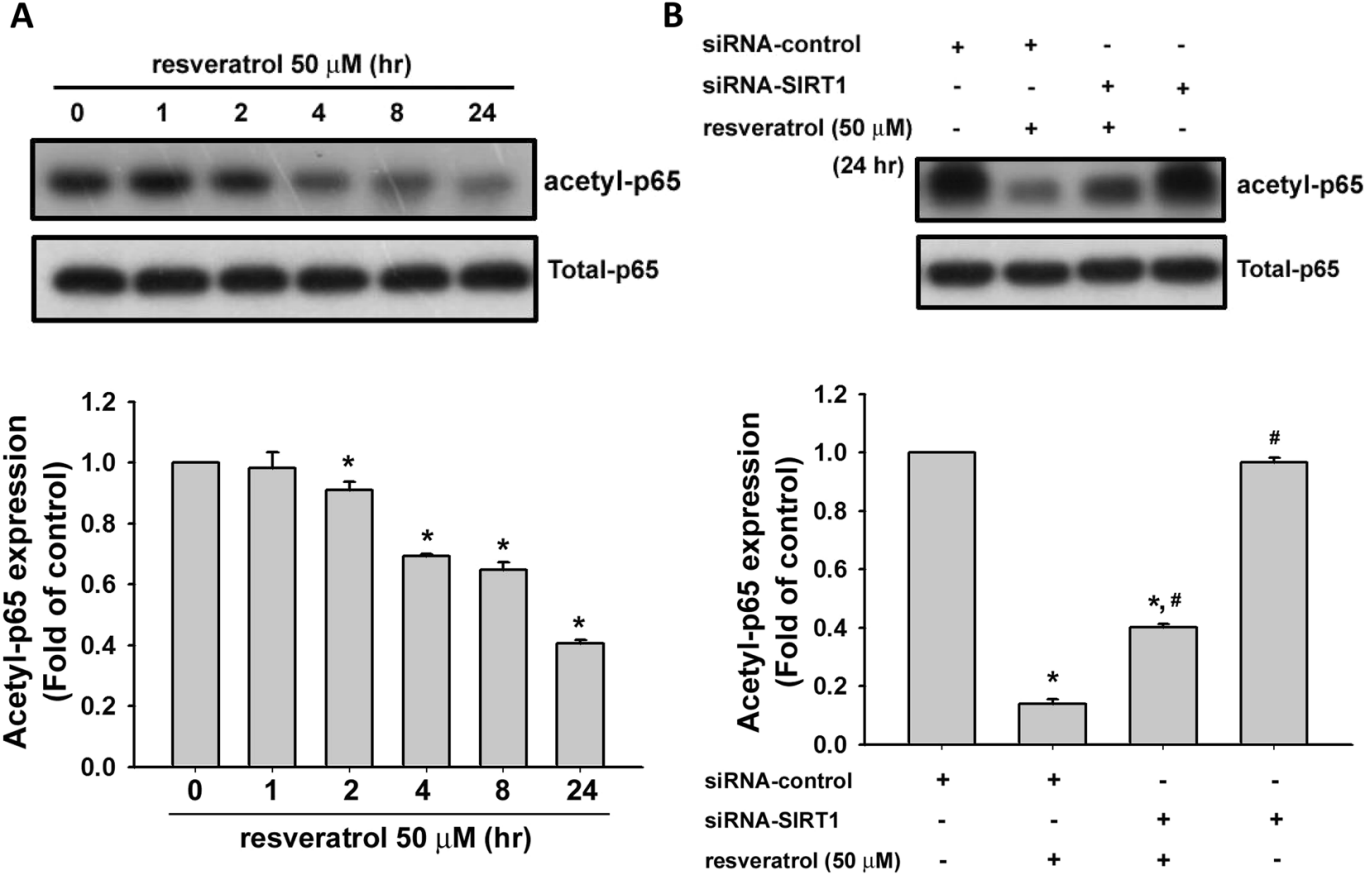
Effect of resveratrol on NF-κB-p65 acetylation in human chondrosarcoma cells. (**A**) JJ012 cells were incubated with resveratrol (50 μM) for various time intervals. The acetylation of NF-κB-p65 expression was examined by Western blot analysis. (**B**) Cells were transfected with SIRT1 or control siRNA for 24 h with or without resveratrol treatment. The acetylation of NF-κB expression was examined by Western blot analysis. Results are presented as the mean ± SEM (n = 5). ^*^ *P* < 0.05 as compared with control; ^#^ *P* < 0.05 as compared with resveratrol-treated group.

Resveratrol has been shown to be as an inhibitor of NF-κB signaling^36^. We next investigated whether SIRT1 activation by resveratrol affected NF-κB signaling in human chondrosarcoma cells. As shown in Fig. 3A, resveratrol (50 μM) significantly reduced the acetylation of NF-κB-p65 in a time-dependent manner. Transfection of cells with SIRT1 siRNA significantly reversed the resveratrol-induced deacetylation of NF-κB-p65 (Fig. 3B).

### Deacetylation inhibitor reduces resveratrol-increased caspase-3 activation

**Figure 4:**
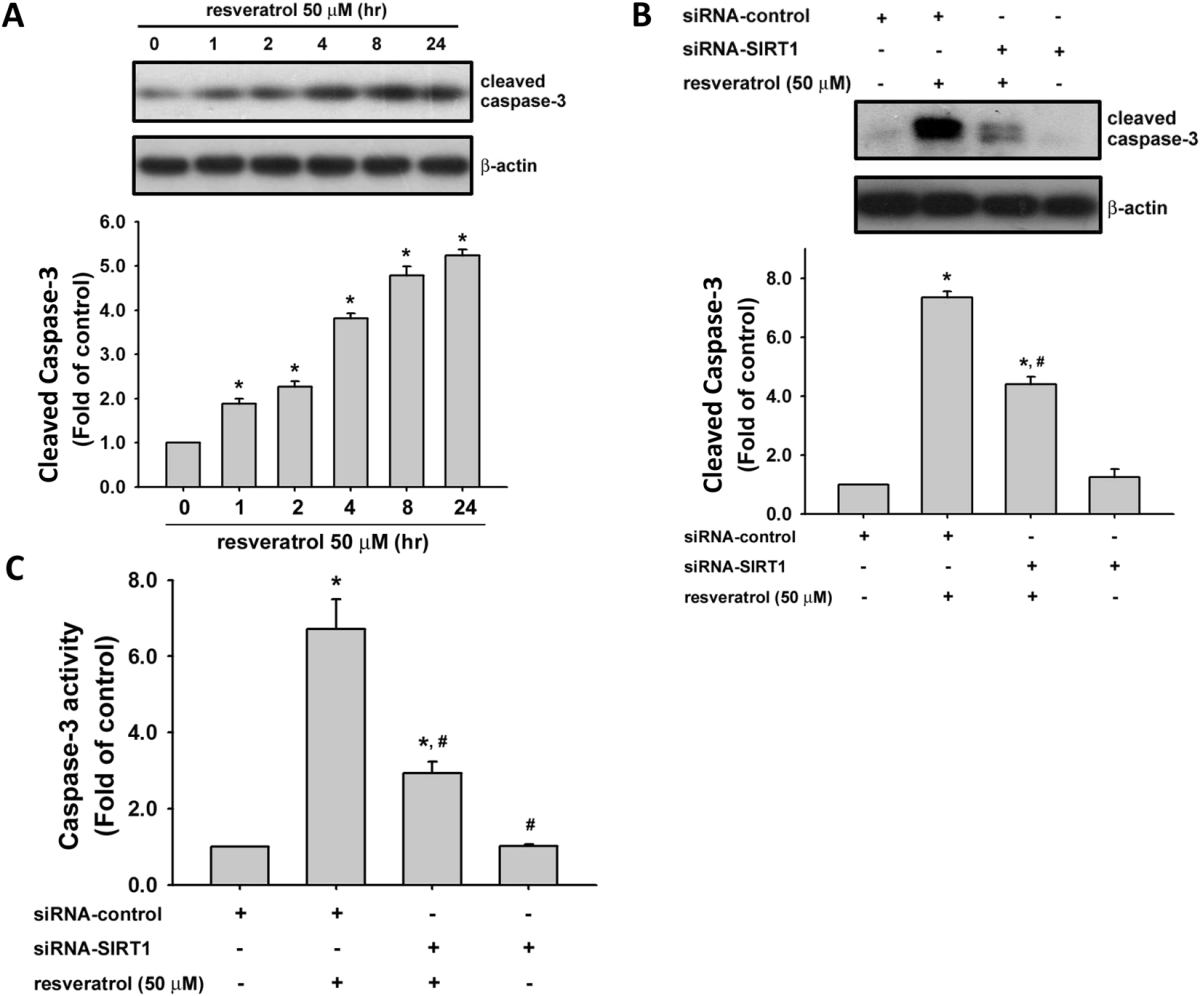
Resveratrol induced the activation of caspase-3 in human chondrosarcoma cells. (**A**) JJ012 cells were incubated with resveratrol (50 μM) for various time intervals. The caspase-3 protein expression was examined by Western blot analysis. (**B**) and (**C**) Cells were transfected with SIRT1 or control siRNA for 24 h with or without resveratrol treatment. The caspase-3 protein expression was examined by Western blot analysis (**B**). The caspase-3 activity was determined by caspase-3 ELISA kit (**C**). Results are presented as the mean ± SEM (n = 5). ^*^ *P* < 0.05 as compared with control; ^#^ *P* < 0.05 as compared with resveratrol-treated group.

**Figure 5:**
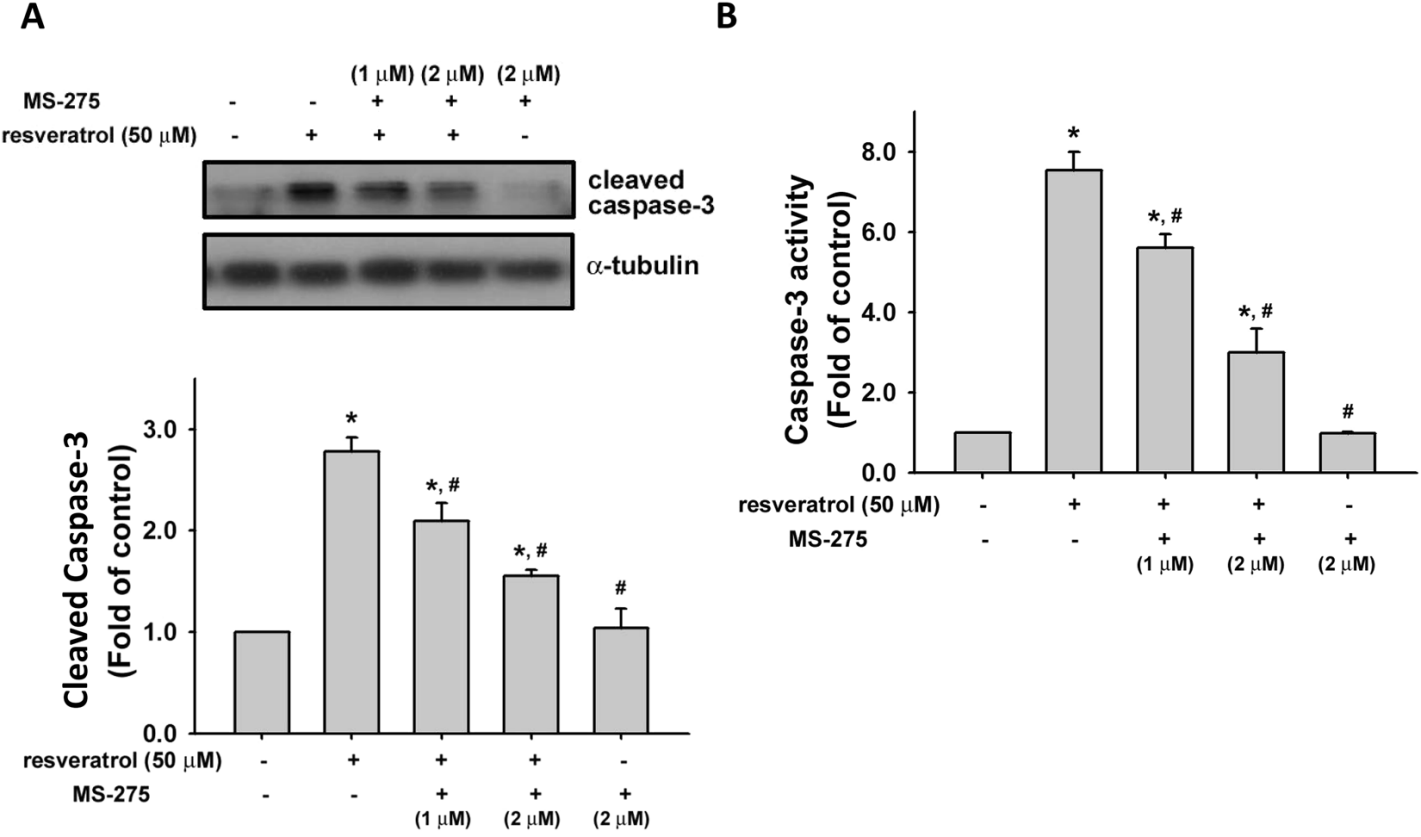
Effect of deacetylation inhibition on resveratrol-increased caspase-3 activation in human chondrosarcoma cells. (**A**) and (**B**) JJ012 cells were pretreated with deacetylases inhibitor MS-275 (1 and 2 μM) for 1 h and then treated with resveratrol (50 μM) for 24 h. The caspase-3 expression was examined by Western blot analysis (**A**). The caspase-3 activity was examined by caspase-3 ELISA kit (**B**). Results are presented as the mean ± SEM (n = 5). ^*^ *P* < 0.05 as compared with control. ^#^ *P* < 0.05 as compared with resveratrol-treated group.

Caspase-3 plays a role in apoptosis to trigger the stages of cell death. We next tested the activated effect of resveratrol on caspase-3 and examined whether deacetylation inhibitor would reverse the effect of resveratrol. As shown in Fig. 4A, resveratrol (50 μM) significantly increased the cleavage of caspase-3 protein in chondrosarcoma cells in a time-dependent manner. Transfection of cells with SIRT1 siRNA significantly reduced the increased cleavage of caspase-3 protein (Fig. 4B) and caspase-3 activity (Fig. 4C) in resveratrol-treated chondrosarcoma cells. Moreover, pretreatment of cells with histone deacetylase inhibitor MS-275^37^ could also significantly reverse the increased cleavage of caspase-3 protein (Fig. 5A) and caspase-3 activity (Fig. 5B) in resveratrol-treated chondrosarcoma cells.

### Resveratrol significantly retards tumor growth in Nu/Nu nude mice xenograft model of JJ012 cells

**Figure 6:**
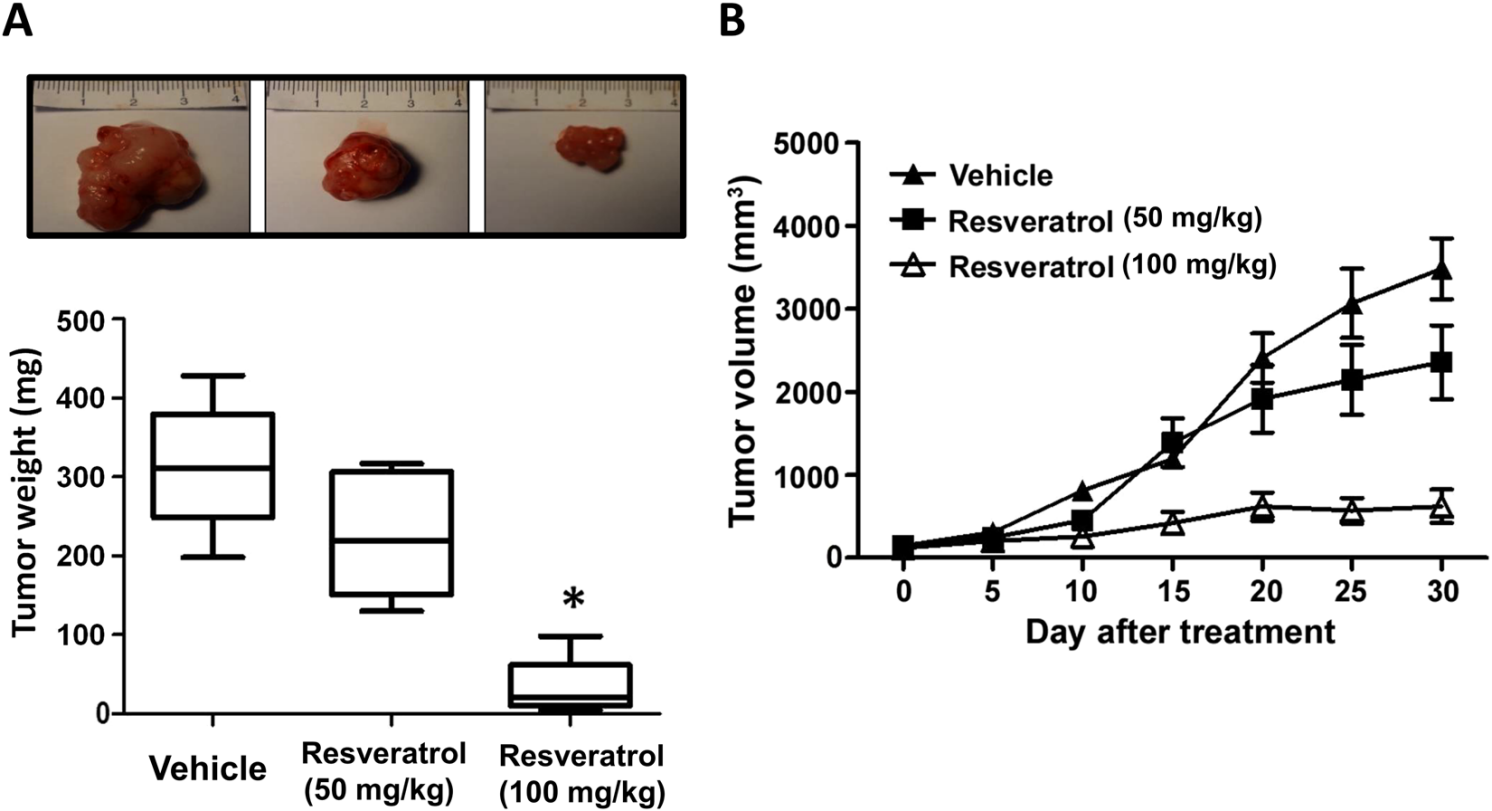
Resveratrol abolished the growth of chondrosarcoma xenografts in mice. Nu/Nu nude mice bearing tumor xenografts were injected intraperitoneally with vehicle (DMSO) and resveratrol (L: 50 mg/kg; H: 100 mg/kg) once per day for 30 days. (**A**) The tumor images represent excised tumors from each group and tumor weights were compared between the resveratrol-treated and control (DMSO) groups on the last day of treatment. (**B**) Tumor volumes measured during the treatment are presented as the responses. Results are presented as the mean ± SEM from ten tumor samples per group. ^*^ *P* < 0.05 as compared with control.

**Figure 7:**
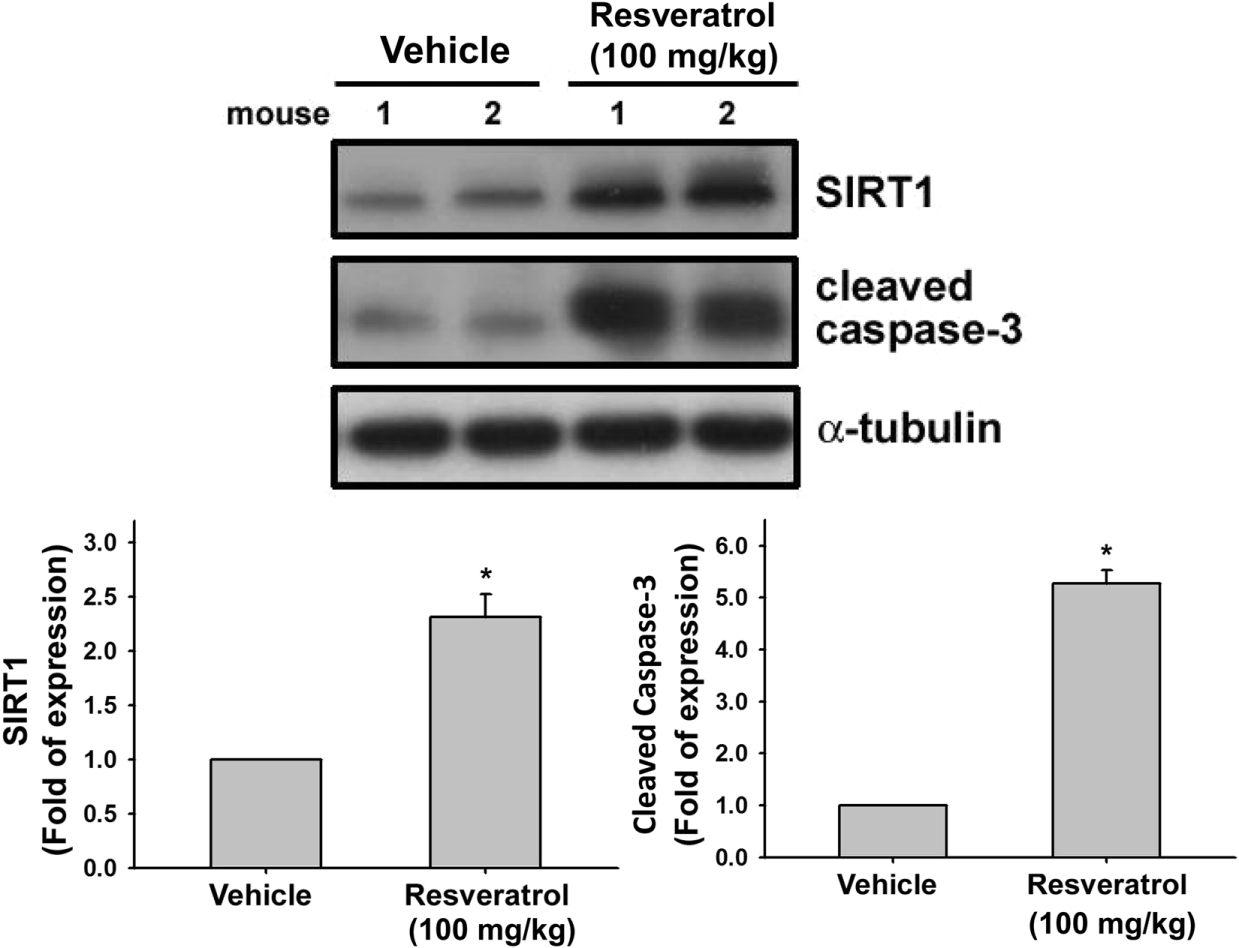
Resveratrol increased the protein expressions of SIRT1 and cleaved caspase-3 in mouse xenograft tumors. Nu/Nu nude mice bearing tumor xenografts were injected intraperitoneally with vehicle (DMSO) and resveratrol (100 mg/kg) once per day for 30 days. The protein expression was determined by Western blotting. Densitometric analyses for SIRT1 and cleaved caspase-3 level corrected to α-tubulin were shown. Results are presented as the mean ± SEM from five tumor samples per group. ^*^ *P* < 0.05 as compared with control.

**Figure 8:**
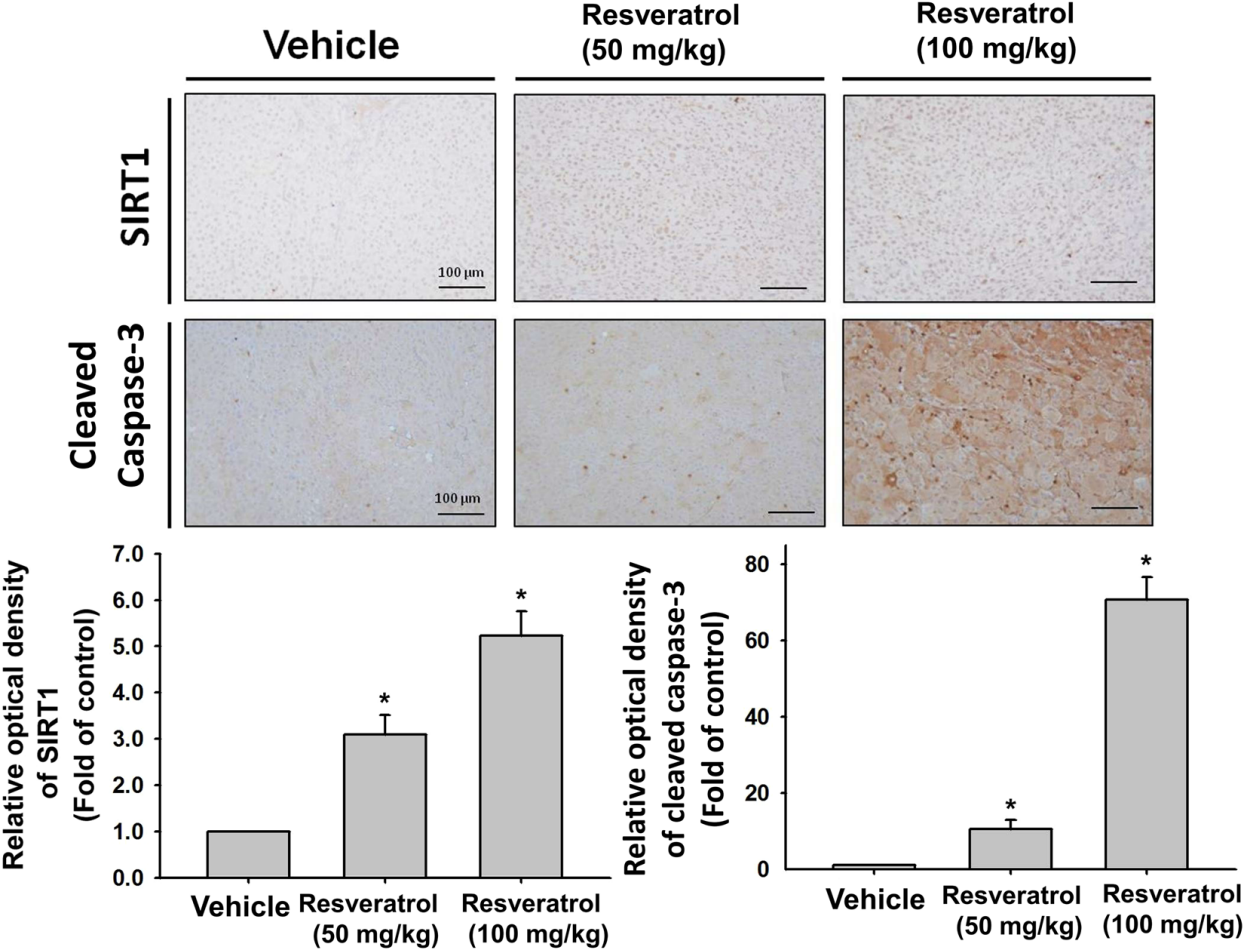
Immunohistochemical changes of SIRT1 and cleaved caspase-3 expressions in tumors of xenograft mouse model with or without resveratrol treatment. Nu/Nu nude mice bearing tumor xenografts were injected intraperitoneally with vehicle (DMSO) and resveratrol (50 and 100 mg/kg) once per day for 30 days. Scale bar = 100 μm. The semi-quantitative analyses of immunohistochemical changes were shown. Results are presented as the mean ± SEM from five tumor samples per group. ^*^ *P* < 0.05 as compared with control.

To verify the *in vitro* effects of the resveratrol-induced apoptotic effect, we decided to determine whether resveratrol possessed anti-tumor activities *in vivo*. We established xenografts of JJ012 cells in nude mice; as tumors reached 100–200 mm^3^ in size, the mice were divided into three groups and treated with either vehicle or resveratrol (50 mg/kg or 100 mg/kg) for 30 days. At the end of the treatment, the tumors in the resveratrol-treated group showed significantly smaller in size and weight than those in the control group (Fig. 6A). Tumor growth was markedly inhibited in the resveratrol-treated group (100 mg/kg), whereas tumors in the control group continued to grow as large as 3500 mm^3^, with weights of 310 mg, respectively (Fig. 6A,B). Moreover, treatment of resveratrol did not affect the body weight of the mice compared with the control group (data not shown), suggesting that resveratrol was not toxic *in vivo*. Moreover, *ex vivo* analysis of tumors excised from mice showed significantly increased SIRT1 and cleaved caspase-3 expressions in the resveratrol-treated group compared with that in the control group, as shown by Western blot (Fig. 7) and immunohistochemistry (Fig. 8). These results indicated that resveratrol suppressed the growth of xenograft tumors in Nu/Nu nude mice, and SIRT1 did play a role in resveratrol-induced chondrosarcoma cell apoptosis.

## Discussion

Chondrosarcoma is a rare but malignant form of bone cancer^1^. Chondrosarcoma is resistant to chemotherapy and radiotherapy due to abundant extracellular matrix, poor vascularity, and low percentage of dividing cells^1^. Unlike osteosarcoma and Ewing’s sarcoma, which are mesenchymal malignancies with dramatic increase in long-term survival after systemic chemotherapy, chondrosarcoma has a high incidence of fatality due to lack of an effective adjuvant therapy^38^. Therefore, it is important to explore novel therapeutic strategies for malignant characterization of chondrosarcoma to improve the prognosis. Resveratrol is a naturally occurring phytoalexin and is known to possess protective potential in several disease models^39, 40^. Much evidence appears that resveratrol has anti-cancer activity and its theoretical molecular targets^41^. Resveratrol at the concentrations of 25–100 μM has been shown to induce chondrosarcoma cell apoptosis^21^. Im *et al*. have found that resveratrol (25 and 50 μM) significantly reduces survival in chondrosarcoma cells, but not primary articular chondrocytes^22^. The detailed mechanisms of resveratrol on chondrosarcoma remain to be clarified. In the present study, the results demonstrate the anti-cancer activity of resveratrol on chondrosarcoma cells in terms of apoptosis induction and growth inhibition *in vitro* and *in vivo*. We showed that resveratrol exerted a marked cell growth inhibition and apoptosis induction at 10–200 μM after 48 h of incubation of chondrosarcoma cells. We also observed that resveratrol at the higher concentration of 200 μM induced not only apoptosis but also necrosis (Fig. 1B, annexin V^−^/PI^+^). Moreover, caspase-3 activation is considered to be the sign of final commitment to cell death and a hallmark of apoptosis. We found that resveratrol time-dependently induced the increase in caspase-3 activity in cultured human chondrosarcoma cells and in tumors of xenograft mice. Therefore, our findings suggest that resveratrol exerts a potential on anti-cancer activity in human chondrosarcoma cells *in vitro* and *in vivo*.

Sirtuins, homologous to yeast silent information regulator 2 (Sir2), is cloned and characterized over the past decades as a gene involved in the epigenetic silencing in yeast and can be found in nearly all species^42^. In mammals, there are seven sirtuins (SIRT1-7) that localize in the nucleus, cytoplasm, or mitochondria. Sirtuins are known to avail different substrates and regulate a broad spectrum of cellular and physiological functions including cell proliferation, differentiation, DNA damage, genome stability, energy homeostasis, organ development, aging, and cancer^27^. Among these seven sirtuins, SIRT1 shared the most homolog of the yeast Sir2 protein in sequence and is the most studied. SIRT1 carries the acetyl group away from the ε-amino group of lysine residues in histones and non-histone proteins, and regulates target gene expression and protein activities that modulate several cellular processes such as cell proliferation, apoptosis, differentiation, metabolism, DNA damage and stress response, genome stability, and cell survival in complex matters^27^. However, the role of SIRT1 in various human tissues and tumor models are still controversial. Upregulation of SIRT1 in breast cancer cells is correlation to inactivation of tumor suppressor HIC1 (hypermethylated in cancer 1) by DNA hypermethylation^43^. SIRT1 inhibits the proliferation of pancreatic cancer cells by expressing oncogenic pancreatic adenocarcinoma upregulated factor (PAUF) and inhibition of β-catenin and cyclin-D1^29^. In hematopoietic progenitor cells, oncogenic breakpoint-cluster-region-Abelson-murine-leukemia (BCR-ABL) can activate SIRT1 through STAT5 signaling, which promotes leukemogenesis^44^. Overexpression of SIRT1 suppresses intestinal tumorigenesis and colon cancer growth by deacetylation of oncogene β-catenin, preventing its localization to the nucleus^45^. Recently, the *in vitro* study of Feng *et al*. demonstrated that the expression of SIRT1 in chondrosarcoma cells could effectively regulate the metastatic plasticity of the cells by inducing epithelial-mesenchymal transition^46^. Jin *et al*. indicated that treatment with SIRT1 agonist resveratrol *in vitro* induced apoptosis, inhibited proliferation, and affected phosphorylation within the STAT3 signaling pathway by activating Sirt1 in SW1353 chondrosarcoma cells^47^. However, the role of SIRT1 in oncogenic and tumor-suppressive function in chondrosaroma still remains to be clarified. Moreover, the therapeutic effect of resveratrol on chondrosarcoma has also not yet been determined in animal model. These prompted us to study the involvement of this key deacetylase, SIRT1, in the resveratrol-related anti-cancer activity in human chondrosarcoma cells. In the present study, we found that resveratrol (5–100 μM) effectively increased the SIRT1 expression and activity in human chondrosarcoma cells. Both SIRT1 siRNA transfection and histone deacetylase inhibitor MS-275 could significantly reverse the resveratrol-increased caspase-3 activity. Both SIRT1 and cleaved caspase-3 expressions were also increased in tumors of resveratrol-treated xenograft mice. These results indicate that resveratrol induce chondrosarcoma cell apoptosis through a SIRT1-regulated signaling pathway. On the other hand, SIRT3 possesses the potential to regulate the cancer processes and may be the therapeutic target for cancer^48^. Resveratrol is also a SIRT3 activator that has been suggested to be the therapeutic potential for cancer prevention in neuroblastoma, hepatoma, breast, lung, pancreatic, and prostate cancers^48^. However, the role of SIRT3 in chondrosarcoma prevention or therapy still remains to be clarified in the future.

It has been demonstrated that both SIRT1 and NF-κB exhibit ancient signaling pathways, which regulate metabolic and inflammatory disorders in mammals through mutually opposing control mechanisms^49^. A previous study has indicated that activation of SIRT1 can attenuate senescence-associated lung inflammation via inhibition of NF-κB signaling^50, 51^. In mammals, the transcription factor family of NF-κB consists of five members, p65 (RelA), RelB, c-Rel, p105/p50 (NF-κB1), and p100/52 (NF-κB2)^52^. Yeung *et al*. have indicated that SIRT1 can deacetylate the RelA/p65 subunit of NF-κB at lysine 310 and its recruitment to the NF-κB sites in chromatin was a promoter-specific event in non-small-cell lung cancer cells^53^. Treatment with the resveratrol (10–50 μM) activated chromatin-associated SIRT1 protein on the cIAP-2 promoter region, which resulted in a loss of NF-κB-regulated gene expression and consequently augmented the tumor necrosis factor α (TNFα)-induced apoptosis in human non-small-cell lung cancer cells^53^. This led us to investigate whether p65 component of the NF-κB complex transcription was regulated by the sirtuin family of NAD^+^-dependent deacetylases in resveratrol-induced apoptosis in human chondrosarcoma cells. In the present study, we found that resveratrol could effectively deacetylate p65 component of the NF-κB complex in human chondrosarcoma cells in a time-dependent manner. Treatment with SIRT1 siRNA could significantly reverse the expression of acetylated p65. These findings suggest that resveratrol induces apoptosis in human chondrosarcoma cells via SIRT1 activation-mediated deacetylation of p65 subunit of NF-κB complex.

In conclusion, we demonstrate for the first time that SIRT1 activation by resveratrol induces apoptosis in human chondrosarcoma cells through the deacetylation of p65-NF-κB protein and exhibits antitumor activity in a chondrosarcoma mouse xenograft model. These findings provide a potential therapeutic strategy against chondrosarcoma.

## Materials and Methods

### Reagents

Mouse monoclonal antibodies specific for α-tubulin and β-actin were purchased from Santa Cruz Biotechnology (Santa Cruz, CA USA). Rabbit polyclonal antibody specific for acetyl-NF-κB (p65) and cleaved caspase-3 were purchased from Cell Signaling (Danvers, MA, USA). Mouse monoclonal antibodies specific for SIRT1 and SIRT1 Human SimpleStep ELISA kit were purchased from Abcam (Cambridge, MA, USA). Caspase activity assay kit was purchased from Promega Corporation (Madison, WI, USA). N-(2-aminophenyl)-4-[N-(pyridine-3yl-methoxy-carbonyl) aminomethyl] benzamide (MS-275) was purchased from Cayman (Ann Arbor, MI). Resveratrol and all other chemicals were obtained from Sigma-Aldrich (St. Louis, MO, USA).

### Cell culture

The human chondrosarcoma cell line (JJ012) was kindly provided by Dr. Sean P. Scully (University of Miami School of Medicine, Miami, FL, USA). Cells were cultured as previously described^54^. The cells were cultured in DMEM/α-MEM supplemented with 10% Fetal Bovine Serum and maintained at 37 °C in a humidified atmosphere of 5% CO_2_.

### Cell viability assay

Cell viability was determined by 3-(4,5-dimethylthiazol- 2-yl)-2,5-diphenyltetrazolium bromide (MTT; Sigma) assay. After treatment of cells with or without resveratrol for 24 and 48 hours, cells were washed with PBS. MTT (0.2 mg/ml) was then added to each well and the mixture was incubated for four hours at 37 °C. Then, culture medium was changed with 150 μL DMSO to dissolve blue formazan crystals. After the mixture was shaken at room temperature for 10 minutes, the absorbance was measured at 550 nm.

### Quantification of apoptosis by flow cytometry

Apoptosis was assessed using annexin V, a protein that binds to phosphatidylserine (PS) residues, which are exposed on the cell surface of apoptotic cells. Cells were treated with resveratrol for various concentrations (10–200 μM). After treatment, cells were washed twice with PBS (pH 7.4), and re-suspended in staining buffer containing 1 μg/ml propidium iodide (PI) and 0.025 μg/ml annexin V-FITC. Double-labeling was performed at room temperature for 10 min in the dark before the flow cytometric analysis. Cells were immediately analyzed using FACScan and the Cellquest program (Becton Dickinson).

### Western blot analysis

Cellular lysates were prepared for protein expression assay. Equal amounts of protein samples (20–40 μg) were resolved on SDS-PAGE and transferred to immobilon polyvinyl difluoride (PVDF) membranes. The blots were blocked with 4% BSA for 1 h at room temperature and then probed with the primary antibodies against SIRT1, acetyl-NF-κB (p65), NF-κB (p65), cleaved caspase-3, α-tubulin, and β-actin overnight at 4 °C. The blots were subsequently incubated with the secondary goat anti-mouse and anti-rabbit antibodies conjugated with horseradish peroxidase for 1 h at room temperature. The blots were visualized by enhanced chemiluminescence using Kodak X-OMAT LS film (Eastman Kodak, Rochester, NY).

### siRNA transfection

The siRNAs against human SIRT1 and control siRNA were purchased commercially from Invitrogen. Cells were transfected with siRNAs (at a final concentration of 60 nM) using RNAimax purchased from Invitrogen according to the manufacturer’s instructions.

### Caspase activity

This assay is based on the cleavage activity of caspase-3 (DEVDase) on the substrate (Ac-DEVD-pNA), and then the p-nitroaniline (pNA) is released from substrate. CaspACE™ Assay kit (Promega Corporation) was used. The cell lysates were prepared and incubated with specific caspase-3 antibody. Immunocomplexes were incubated with peptide substrate in assay buffer (100 mM NaCl, 50 mM 4-(2-hydroxyethyl)-1-pipera-zine-ethanesulphonic acid (HEPES), 10 mM dithiothreitol, 1 mM EDTA, 10% glycerol, 0.1% 3-[(3-cholamidopropyl)dimethylammonio]-1- propanesulfonate (CHAPS), pH 7.4) for 2 h at 37 °C. The p-nitroaniline release was monitored at 405 nm. Results are represented as the fold change of the activity compared to the control group.

### SIRT1 activity

SIRT 1 activity was determined with SIRT1 Deacetylase Fluorometric Assay kit. Nuclear proteins were extracted using the Nuclear and Cytoplasmic Protein Extraction kit according to the manufacturer’s instructions. The resulting fluorescence was measured at 340 nm excitation and 440 nm emission wavelengths with a fluorescent microplate reader.

### *In vivo* tumor xenograft study

This animal study was approved and conducted by the institutional animal care and use committee, College of Medicine, National Taiwan University. All experiments involving animals complied with the ARRIVE guidelines. To develop xenograft tumors, mice were treated humanely and with regard for alleviation of suffering. Male nude mice [6 weeks old; BALB/cA-nu (nu/nu)] were purchased from National Laboratory Animal Center. Mice were housed five per cage under standard laboratory conditions at a constant temperature 22 ± 2 °C with 12-h light/dark cycles. To develop xenograft tumors, a total of 1 × 10^6^ cells were suspended in 200 μl of serum-free media and mixed with an equal volume of Matrigel (BD Biosciences, Bedford, MA, USA). The mixtures were injected subcutaneously into the dorsal flanks of 8-week-old Nude mice. After the tumors had grown to approximately 100–200 mm^3^ (around 21 days), treatment was initiated. The mice were intraperitoneally injected with vehicle, 50 or 100 mg/kg resveratrol every day for 30 days (5 mice / group). The volume of the implanted tumor in dorsal side of mice was measured with a caliper, using the formula volume = longest tumor diameter × (shortest tumor diameter)^2^/2. After 35 days of treatment, mice were sacrificed, and the tumor tissues were collected for further analyses.

### Immunohistochemistry

Tumor samples were fixed in PBS containing 4% paraformaldehyde at 37 °C for 24 h, decalcified in 10% Na 2 EDTA (pH 7.6) at 4 °C for 2 weeks and then dehydrated in increasing concentrations of ethanol. Serial sections with a thickness of 5 μm were taken. The sections were treated with 3% H_2_O_2_ in methanol and then incubated with protease type XIV at 37 °C. The sections were blocked with 10% BSA containing 1% Triton X-100 and then incubated with anti-SIRT1 and caspase-3 at 4 °C overnight. The sections were subsequently incubated with secondary antibodies and then with avidin–biotin–peroxidase complex (Vector Laboratories). Finally, the labeling was revealed by treatment with 0.01% H_2_O_2_ and 0.05% diaminobenzidine. Relative optical density of images was semi-quantified using ImageJ analysis software with Immunohistochemistry Image Analysis Tool box (v. 1.48; National Institutes of Health).

### Statistics

The results are presented as mean ± SEM. Each experiment was performed four times to ensure reproducibility. The significant difference from the respective controls for each experimental test condition was assessed by one-way analysis of variance (ANOVA) and Dunnett test. When the *P*-value is less than 0.05, the difference is significant. Software used: SigmaPlot 10.0 and GraphPad Prism 5.
